# NKT cells are responsible for the clearance of murine norovirus through the virus-specific secretory IgA pathway

**DOI:** 10.1016/j.bbrep.2019.100722

**Published:** 2020-01-02

**Authors:** Hiroki Ishikawa, Satoshi Ino, Toshiko Yamochi, Hiraku Sasaki, Takahiro Kobayashi, Chikara Kohda, Masafumi Takimoto, Kazuo Tanaka

**Affiliations:** a Department of Microbiology and Immunology, Showa University School of Medicine, Shinagawa-ku, Tokyo, 142-8555, Japan; b Department of Pathology and Laboratory Medicine, Showa University School of Medicine, Shinagawa-ku, Tokyo, 142-8555, Japan; c Department of Health Science, Juntendo University School of Health and Sports Science, Inzai, Chiba, 270-1695, Japan

**Keywords:** Murine norovirus-S7, NKT cells, Secretory IgA, Delayed growth

## Abstract

Norovirus infection cause epidemic nonbacterial gastroenteritis in patients. The immune mechanisms responsible for the clearance of virus are not completely understood. We examined whether NKT cells are effective against norovirus infection using CD1d KO mice. The body weights of 4-weeks-old CD1d KO mice that were infected with murine norovirus-S7 (MNV-S7) were significantly lower than those of non-infected CD1d KO mice. On the other hand, the body weights of infected WT mice were comparable to those of non-infected WT mice. Correspondingly, CD1d KO mice had an almost 1000-fold higher MNV-S7 burden in the intestine after infection in comparison to WT mice. The mechanism responsible for the insufficient MNV-S7 clearance in CD1d KO mice was attributed to reduced IFN-γ production early during MNV-S7 infection. In addition, the markedly impaired IL-4 production in CD1d KO mice resulted in an impaired MNV-S7-specific secretory IgA production after MNV-S7 infection which is associated with mucosal immunity. Thus, the present results provide evidence that NKT cells play an essential role in MNV-S7 clearance.

## Highlights

• This is the first report that NKT cell is responsible for protection against MNV infection.
• The growth curves of infected CD1d KO mice were significantly lower than non-infected CD1d KO mice.
• MNV titers in fecal of CD1d KO mice were almost 1000-fold more than that of WT mice after infection.
• MNV-specific sIgA production in CD1d KO mice was significantly lower resulting in insufficient MNV clearance.

## 1 Introduction

Human norovirus infection is the leading cause of seasonal epidemic non-bacterial gastroenteritis in patients of all ages. Norovirus is transmitted via the consumption of contaminated food, and as a secondary infection via person-to-person infection. Patients infected with norovirus show various gastrointestinal symptoms, including nausea, vomiting and diarrhea, which typically resolve within 1–4 days. In the United States, 20,000 patients are infected with norovirus every year, nearly 800 of whom die [1]. Unfortunately, there are no vaccines or effective therapeutic materials for norovirus.

Murine norovirus (MNV)-1 was originally isolated from the brain tissue of *Rag*^−/-^/*Stat1* mice [2]. Although MNV is quickly cleared in WT mice, the viral infection is lethal in *Stat1*^−/−^ mice, which cannot mediate IFNs-stimulated gene transcription signal pathway involving IFN-α, IFN-β and IFN-γ, in association with a high MNV titer burden in multiple tissues [2,3], We also showed that IFN-α/β induced by lactoferin prevent MNV-S7 replication in vitro [4]. These reports imply that the IFNs signal pathway plays a major role in MNV clearance. Moreover, both IFN-γ and IFN-α/β play important roles in the clearance of MNV, because mice with the deletion of both IFN-γ receptor and IFN-α/β receptor are more susceptible to MNV-related mortality than mice in which either IFN receptor is deleted individually [2,5]. Furthermore, IFN-γ can induce autophagy and transcription factor IRF-1, which incline the Th1/Th2 balance toward a Th1 immune response, resulting in the suppression of MNV replication in macrophages [[5], [6], [7]]. Pretreatment of IFN-γ in cultured macrophages and dendritic cells blocks the translation of viral protein without the degradation of viral genomes [8]. IFN-γ is thus responsible for protection against MNV infection, although IFN-γ alone is inadequate for achieving complete MNV clearance [9].

Accumulating reports indicate that NKT cells play an important role in protecting the host against infection with many kinds of pathogens, including bacteria, viruses, parasites and fungi [10,11]. NKT cells can immediately secrete a large amount of IFN-γ after activation. This is followed by stimulation of innate and adaptive immunity, involving NK cells, T cells, B cells and dendritic cells [12,13]. We previously have reported that NKT cell activation induces an increase in the cytotoxic activity of NK cells and in the killing activity of antigen-specific CD8^+^ T cells during influenza virus infection in an IFN-γ-dependent manner, leading to influenza virus clearance and the improvement of the clinical condition [14].

The effects of NKT cells against MNV-S7 infection remain unclear. In the current study, we evaluate whether NKT cells are involved in the clearance of the viral load and the pathogenesis of MNV-S7 using NKT-deficient mice.

## 2 Materials and methods

### 2.1 Virus and virus titer

Murine norovirus-S7 (MNV-S7) was kindly provided by Dr. Kyuwa at the University of Tokyo, who determined the genome sequence (GeneBank accession No. AB435515) [15]. MNV-S7 was cultured with confluent Raw 264.7 cells at 37 °C for 3 days and the cultured supernatants, including progeny MNV-S7, were collected. Then, the supernatants were filtered by a 0.22-μm filter unit. Aliquots of virus fluid were frozen at −80 °C until use. MNV-S7 titers were determined by the TCID_50_ method as described previously [4]. In some experiments, MNV-S7 titers were measured by a quantitative real-time PCR referring to standard cDNA generated from MNV-S7 RNA, which is determined based on the virus titer obtained by the TCID_50_ method [4].

### 2.2 Animals and MNV-S7 infection

Female Balb/c mice as wild-type (WT) mice were purchased from Oriental yeast Co., Ltd. (Tokyo, Japan). CD1d knockout (KO) mice, bred on a Balb/c background, were bred in our experimental animal facility; female mice were used in the present study. The mice were housed under specific pathogen-free conditions according to the animal protocol guidelines of the Institutional Animal Care and Use Committee of Showa University (Protocol No. 08089 and 09004). Balb/c mice or CD1d KO mice (4 weeks of age) were orally infected with 5 × 10^5^ pfu/mouse of MNV-S7. The day of infection was defined as day 0 through the experiments. The body weights of the mice were monitored daily until 28 days after MNV-S7 infection. The weight of each mouse on day 0 before infection was considered to be 100%.

### 2.3 The fecal MNV-S7 titer

Balb/c mice or CD1d KO mice (4 weeks of age) were orally infected with 5 × 10^5^ pfu/mouse of MNV-S7 and feces were collected at days 1, 3, 5 and 7 after MNV-S7 infection. To determine the MNV-S7 titer in feces by a quantitative real-time PCR, viral RNA were extracted using a MagMax viral RNA isolation kit (Ambion, TX, USA), then cDNAs were synthesized using a High capacity RNA-to-cDNA kit according to the manufacturer's instructions (Applied Biosystem, CA, USA). The sequences of probe and primers used in the quantitative real-time PCR were described in a previous study [4]. The quantitative real-time PCR was performed using an ABI PRISM 7000 sequence detection system with the included software program.

### 2.4 Total and MNV-S7-specific secretory IgA production in the small intestine

To measure the amount of total secretory IgA and MVV-S7-specific secretory IgA in the small intestine, the small intestines were excised from each mouse and the contents were washed out with 1 ml of PBS on days 1, 3, 5 and 7 after infection. The washed contents were then centrifuged, and the supernatants were used for measurement. The total secretory IgA concentration in the supernatants was measured using a mouse IgA ELISA quantitation kit (Bethy Laboratories Inc, TX, USA), according to the manufacturer's instructions. To determine the MNV-S7-specific secretory IgA concentration, an ELISA plate was coated with 1 μg/ml (equivalent to 1 × 10^7^/ml of MNV) of MNV-S7, which was washed with PBS by ultra-centrifugation, in 0.1 M NaHCO_3_. After blocking with 1% BSA solution, the supernatants were applied and incubated. HRP conjugated goat anti-mouse IgA (Bethy Laboratories Inc, TX, USA) was added, then an enzymatic color reaction was initiated after washing plate followed by the addition of stop solution. The absorbance at 450 nm is shown in Fig. 2C.

### 2.5 Cytokine production in mesenteric lymph node (MLN) cells

To measure the cytokine production of MLN cells, MLNs were removed from mice, in non-infected mice and at days 1, 3, 5 and 7 after viral infection. MLN cells were plated at 2.5 × 10^5^ cells/ml in a 96 well U-bottom plate stimulated with plate-bound 1 μg/ml anti-CD3 antibody (clone: 2C11) in DMEM containing 10% FBS. After culturing for 48 h, the supernatants were collected to measure the IFN-γ and IL-4 levels by ELISA methods (R&D systems, MN, USA).

### 2.6 Histopathology of the small intestine

For the histopathological examination of the small intestine, in non-infected mice and at days 1, 3 and 10 after viral infection, the ileum region of the small intestine was removed from each mouse and fixed with 10% formaldehyde solution. Then, the paraffin-embedded tissues were cut by a microtome. The sections were stained with hematoxylin-eosin (HE).

### 2.7 Statistical analyses

The statistical analyses were performed using the Excel software program (Microsoft, WA, USA). In all statistical analyses, the statistical significance of the findings was determined using the unpaired *t*-test. P values of less than 0.05 were considered to indicate statistically significance. All values in the figures are presented as the mean ± standard deviation.

## 3 Results

### 3.1 MNV-S7 infection at young age causes delayed growth in CD1dKO mice but not WT mice

**Fig. 1:**
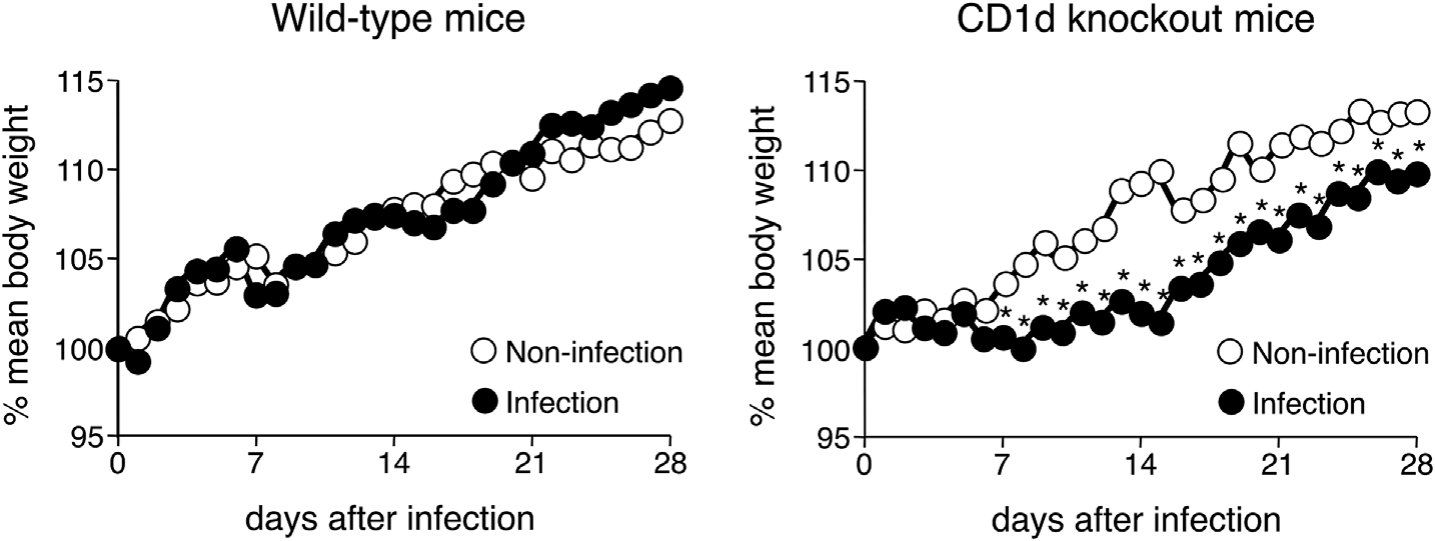
**The body weight curves of WT mice and CD1d KO mice after MNV-S7 infection.** WT mice and CD1d KO mice were orally infected with MNV-S7. Subsequently, the body weights of the mice were monitored daily until day 28. Nine of the WT mice and eight mice of the CD1d KO mice were evaluated for each group. *; p < 0.05 in comparison to the body weight of non-infected mice on the same day. Similar results were obtained in three independent experiments.

To evaluate whether NKT cells have a protective effect against MNV-S7 infection, both WT mice and CD1d KO mice were orally infected with 5 × 10^5^ pfu/mouse of MNV-S7 at 4 weeks of age. Thereafter, their body weights were monitored. Neither of the mouse strains infected with MNV-S7 developed diarrhea by simple visual monitoring or died during the experiment. As shown in Fig. 1, when WT mice were infected with MNV-S7, the body weights increased during the experiment and the growth curve of infected WT mice was comparable to that of non-infected WT mice. On the other hand, the body weights of CD1d KO mice infected with MNV-S7 were significantly lower in comparison to non-infected CD1d KO mice from day 7 to day 28. Interestingly, when WT mice or CD1d KO mice were infected with MNV-S7 at 8 weeks of age, their body weight curve was comparable to that of non-infected WT mice or CD1d KO mice, respectively (data not shown). These results suggest that MNV-S7 infection of 4-weeks-old CD1d KO mice caused growth delay in comparison to non-infected CD1d KO mice.

### 3.2 The MNV-S7 clearance function in the intestine of CD1d KO mice is impaired in comparison to WT mice

**Fig. 2:**
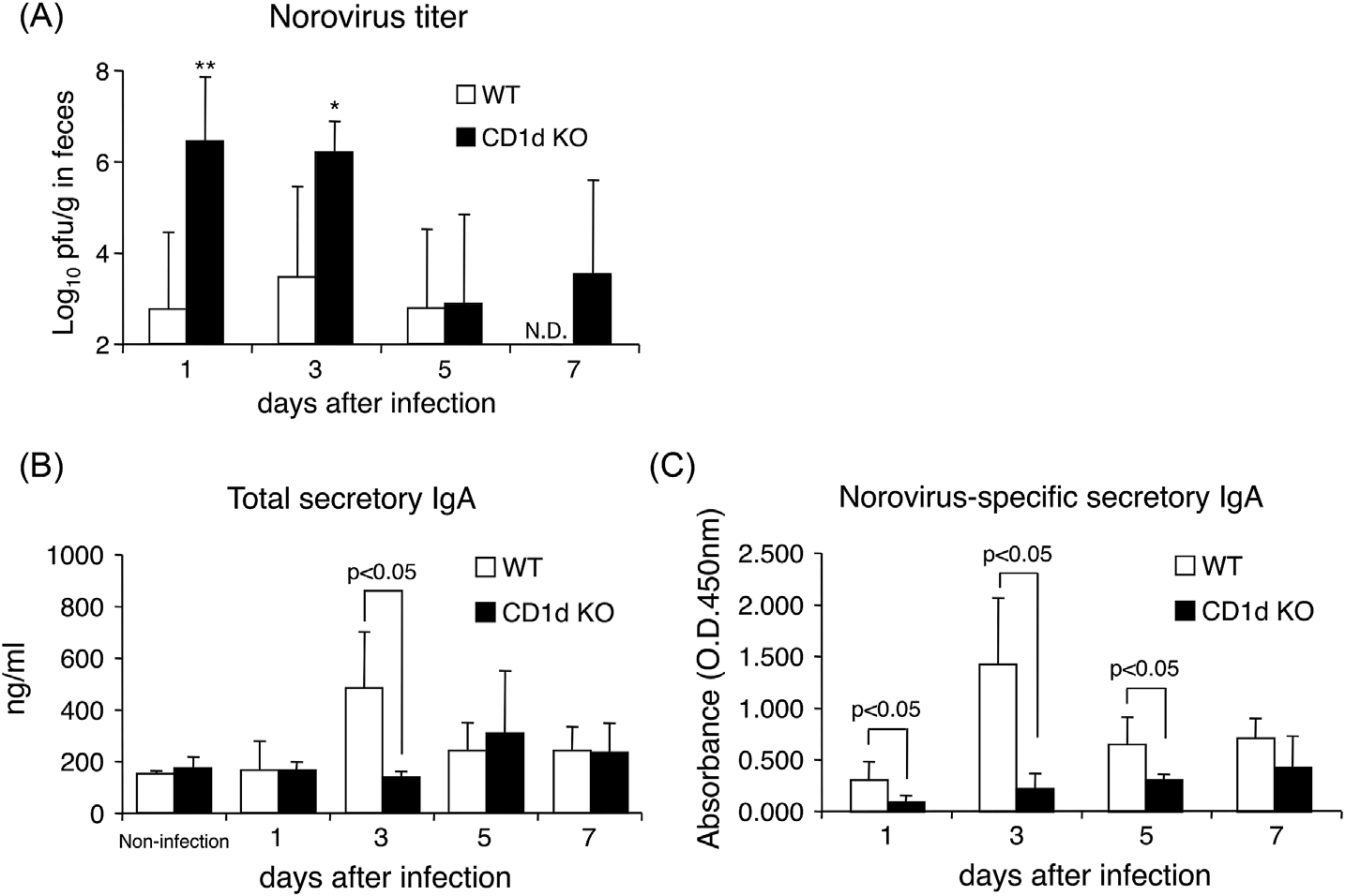
**The fecal MNV-S7 titers and the total and MNV-S7-specific secretory IgA production of WT mice and CD1d KO mice after MNV-S7 infection.** WT mice and CD1d KO mice were orally infected with MNV-S7. (A) The feces of mice were collected to measure the MNV-S7 titers by a quantitative real-time PCR on days 1, 3, 5 and 7 after MNV-S7 infection. The washed contents of the small intestine were collected on days 1, 3, 5 and 7 after infection. ELISA was performed to measure the total (B) and MNV-S7-specific secretory IgA (C). **; p < 0.01, *; p < 0.05 in comparison to the MNV-S7 titer of WT mice. Five mice were used in each group.

To estimate the MNV-S7 clearance function in the intestine of WT mice and CD1d KO mice, the fecal MNV-S7 titer was measured on days 1, 3, 5 and 7 after MNV-S7 infection. As shown in Fig. 2A, the fecal MNV titers of CD1d KO mice on days 1 and 3 were almost 1000-fold more than the fecal MNV-S7 titers WT mice. Moreover, whereas fecal MNV-S7 titers of WT mice were below the limit of detection until day 7, the fecal MNV-S7 titers of CD1d KO mice were still high on day 7. These results indicate that the MNV-S7 clearance of the small intestine was impaired in CD1d KO mice, which allowed for prolonged MNV infection in comparison to WT mice.

### 3.3 Secretion of MNV-S7-specific secretory IgA in the small intestine of CD1d KO mice after MNV-S7 infection was impaired in comparison to WT mice

As antigen-specific secretory IgA is considered to be responsible for pathogen clearance in the small intestine, MNV-S7-specific secretory IgA production in the small intestine was measured in both mouse strains after MNV-S7 infection. As shown in Fig. 2B, the total secretory IgA concentration in the small intestine was equal between non-infected mice and MNV-S7-infected mice on day 1 in both WT and CD1d KO groups. The total secretory IgA production in CD1d KO mice was not significantly increased in comparison to WT mice on day 3 after viral infection. Moreover, the MNV-S7-specific secretory IgA production in CD1d KO mice was not significantly enhanced in comparison to that of WT mice on days 1, 3 and 5 (Fig. 2C), suggesting that impaired MNV-S7-specific secretory IgA production in the small intestine of CD1d KO mice leads to insufficient MNV-S7 clearance in comparison to WT mice.

**Fig. 3:**
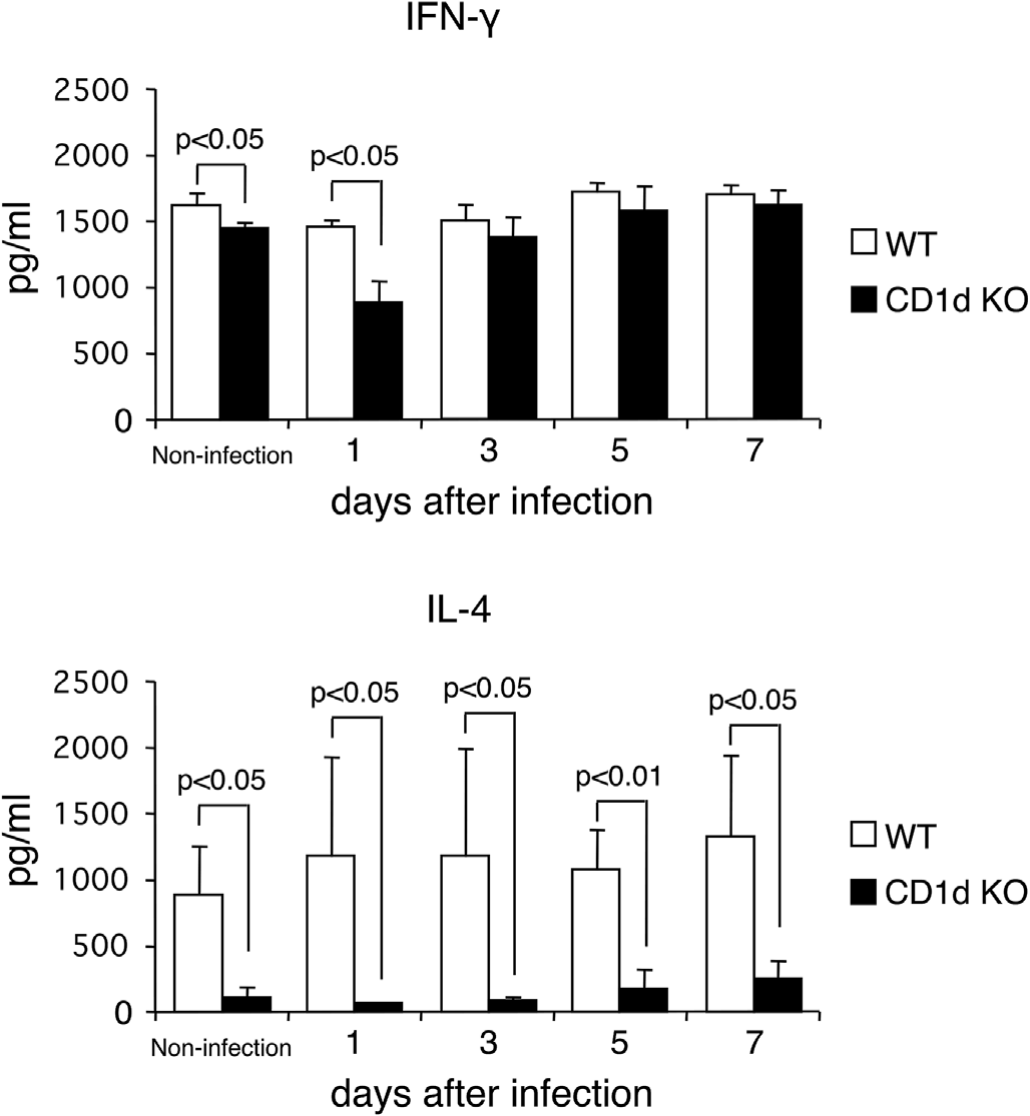
**The IFN-γ and IL-4 production by MLN cells from WT mice and CD1d KO mice infected with MNV-S7.** WT mice and CD1d KO mice were orally infected with MNV-S7. On days 1, 3, 5 and 7 after MNV-S7 infection, MLN cells were cultured with anti-CD3 antibody for 48 h and the cytokines in the culture supernatant were measured by an ELISA. Five mice were used in each group.

### 3.4 The IFN-γ and IL-4 production by MLN cells from CD1d KO mice after MNV-S7 infection is decreased in comparison to that by MLN cells from WT mice

As shown in Fig. 3, the production of IFN-γ by MLN cells from CD1d KO mice, in non-infected mice and on day 1 was significantly reduced in comparison to that of WT mice, but not days 3, 5 and 7 after MNV-S7 infection, suggesting that impaired IFN-γ production of CD1d KO mice may cause high burden of MNV-S7 in early infection. Of note, the production of IL-4 by MLN cells from CD1d KO mice was almost 10-times less than that of WT mice in non-infected mice and on days 1, 3, 5 and 7 after MNV-S7 infection. These results indicated that the MLN cells from CD1d KO mice could not produce cytokine of IL-4 promptly and/or in sufficient amounts in comparison to the MLN cells of WT mice after MNV-S7 infection.

### 3.5 Small intestine tissue damage during MNV-S7 infection was not observed in the histopathological examination of specimens from CD1d KO mice

**Fig. 4:**
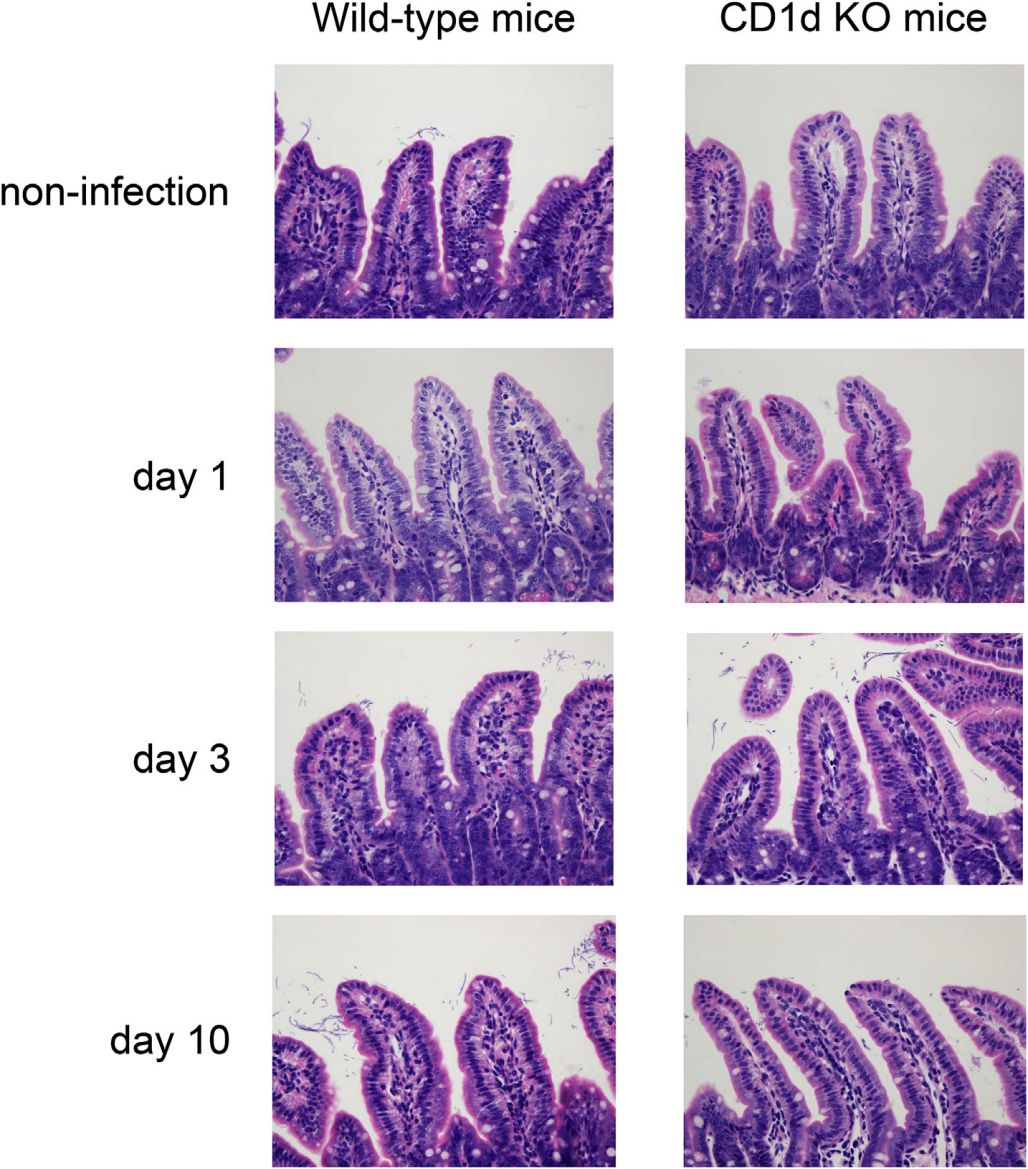
**The histopathology of the small intestine of WT mice and CD1d KO mice with and without MNV-S7 infection.** WT mice and CD1d KO mice were orally infected with MNV-S7. The small intestine was removed from both strains of mice without infection and on days 1, 3 and 10 after infection. Small intestine specimens were fixed with 10% formaldehyde solution, then subjected to HE staining. Observation was performed under 400-fold magnification. Representative images of each intestine section are shown (HE staining).

We next examined whether the small intestinal tissue of CD1d KO mice, which include epithelial cells, villi and micro villi, showed any damage or inflammation caused by MNV-S7 infection, because CD1d KO mice showed delayed growth during MNV-S7 infection. As shown in Fig. 4, no disruption or inflammation was observed in the small intestines of CD1d KO mice or WT mice during MNV-S7 infection.

## 4 Discussion

The purpose of this study was to evaluate whether NKT cells are responsible for protection against MNV-S7 infection and the improvement of the host clinical condition during MNV infection using CD1d KO mice, which is an NKT cell-deficient mouse. To our knowledge, this is first report discussing the interactions between norovirus infection and NKT cells. We demonstrated that CD1d KO mice infected with MNV-S7 showed low body weight in comparison to non-infected CD1d KO mice. The high MNV-S7 burden, which was present for long period in CD1d KO mice may contribute to the impairment of the IFN-γ production in early infection and the MVN-S7-specific secretory IgA production.

The low body weight curve observed in CD1d KO infected with MNV-S7 was only observed when CD1d KO mice were infected with MNV-S7 at a young age (4 weeks of age) and was not observed in adult age (8 weeks of age). This may be because NKT cells may play an important role against pathogenic infection in young mice with less development of innate and adaptive immunity, making young CD1d KO mice more susceptible to MNV infection. Furthermore, because the intestinal microbiota of young age mice are lower in number and/or show less variety in their composition in comparison to adult mice [16], Thus, the maturated microbiota composition in adult mice may also prevent MNV from attaching to intestinal epithelial cells in a competitive manner.

It is known that the histo-blood group antigen or additional carbohydrate moieties, including sialic acid residues on intestinal epithelial cells, work as an MNV receptor [[17], [18], [19], [20]]. However, a study using genome-wide CRISPR/Cas9 guide RNA library recently demonstrated that murine CD300lf and Cd300ld are functional receptors for MNV [21]. CD300lf and CD300ld are mainly expressed on immune cell, such as myeloid cells as granulocytes, bone-marrow mast cell and dendritic cells [22]. The predominant targets of acute MNV infection are therefore reportedly immune cells, including macrophages, dendritic cells, B cells and T cells, within the gut-associated lymphoid tissue (GALT) [[23], [24], [25], [26]]. Furthermore, immune cells in Peyer's patches are a major target of MNV infection, as the MNV production in *Rag 1*^−/−^ mice, which lack Peyer's patches, was found to be significantly reduced compared to that in WT mice [23].

In spite of the lower body weights of MNV-S7 infected CD1d KO mice, no epithelial cell injury or villi or microvilli regression was noted in the small intestine (Fig. 4). Thus, other pathogeneses, such as nutritional malabsorption and/or protein loosing, might account for the lower body weights of MNV-infected CD1d KO mice. More detailed analytical methods, such as electron microscopy may therefore be needed. Another possible explanation for the low body weight curve in CD1d KO mice infected with MNV is that their microbiota composition is altered after MNV infection [27,28]. Hickman et al. reported that MNV infection causes changes in the composition of microbiota, which is similar to the composition of the malnutrition-associated microbiota, however, it is unclear if this is cause or effect of malnutrition [28].

Secretory IgA is the predominant class of antibody in mucosal surface sites, such as the intestine. Notably, antigen-specific secretory IgA production is responsible for preventing and removing toxins and intestinal pathogens, including but not limited to *Salmonella enterica*, *Shigella* species, *Escherichia coli* O157:H7, rotavirus [29]. The protective effects of norovirus-specific secretory IgA against norovirus infection have been reported in humans. Using Norwalk virus-challenged volunteers, Lindesmith et al. reported that Norwalk virus-specific mucosal IgA increase is a good predictor of the risk of Norwalk virus infection among susceptible volunteers [30]. Moreover, norovirus challenge of human volunteers, which found that the number of pre-challenge IgA-producing memory B cells was significantly correlated with protection against infection [31,32]. On the other hand, it has been previously reported that invariant NKT cells induced the release of IgA by B cells without needing invariant NKT cell agonist ligand α-galactosylceramide [33]. In the current study, although impaired IFN-γ production of CD1d KO mice in early infection may cause high burden of MNV-S7, we also showed that the MNV-S7-specific secretory IgA production was impaired in CD1d KO mice infected with MNV-S7 resulting in a high virus burden and delayed virus clearance in comparison to WT mice infected with MNV. As shown in Fig. 2, the MNV-S7-specific secretory IgA production of WT mice on day 1 after infection was not considered to be due to adaptive immune response induction, as it occurred too rapidly to be due to an adaptive immune response, and total secretory IgA concentration on day 1 after infection was comparable to that in non-infected mice. Therefore, the MNV-S7-specific IgA on day 1 after infection may be due to a non-specific cross-reaction against MNV antigen by natural secretory IgA. In contrast, the MNV-S7-specific secretory IgA on day 3 after infection of WT mice may be MNV-S7-specific IgA induced by an adaptive immune response, as the total secretory IgA concentration of WT mice on day 3 after infection were also increased. Furthermore, when mice were infected with MNV via peroral route, MNV infects with macrophages and dendritic cells, B cells and T cells in the sub-epithelial dome of Peyer's patches, which is a major site for serial interaction B cells and dendritic cell that play a critical role in induction of secretory IgA in mucosal [34], and MNV replication was detected within 24 h after infection by *in situ* hybridization using minus-strand MNV RNA [23]. Therefore, mucosal adaptive immunity involving MNV-S7-specific IgA production may be induced earlier than normal immune responses. However, the possibility of increased non-specific cross-reaction against MNV antigen by increased natural IgA production cannot be ruled out. Thus, MNV-S7-specific secretory IgA production is significantly responsible for preventing MNV-S7 infection at the early stage of infection. The impaired MNV-S7-specific secretory IgA production in infected CD1d KO mice might be attributed to the remarkable reduced IL-4 production, because IL-4 and IL-5 work synergistically in secretory IgA production in mucosal immunity [35] In addition, both the secretory component expression and the binding of polymeric IgA on epithelial cells was synergistically enhanced by both IL-4 and IFN-γ [36]. Thus, the impaired production of IL-4 resulted in reduced MNV-specific IgA.

Many reports have demonstrated that NKT cells secrete large amounts of IFN-γ, and that this is followed by the stimulation of various immune responses including innate and adaptive immunity. In a mouse model of influenza virus A infection, we previously reported that the IFN-γ production by activated NKT cells enhanced both NK cells activity and antigen-specific CD8 T cells to eliminate the influenza virus [14]. In a MNV infection model, it was reported that MNV-1 infection was lethal in signal transducer and activator of transcription 1 (*Stat 1*)-deficient mice, in which the IFN family pathway was completely depleted [2]. In this study, the IFN-γ production by MLN cells from infected CD1d KO mice was significantly reduced compared to that from infected WT mice on day 1 after infection resulting in high viral burden of CD1d KO mice. On the other hand, the IL-4 production was remarkably diminished in CD1d KO mice. Similarly, it has been reported that approximately 60% of NKT cells in MLN produced IL-4 upon PMA/ionomycin stimulation, whereas only 1% of the cells produced IFN-γ [37]. Thus, in our infection model, IL-4 seemed to be more crucial than IFN-γ.

In conclusion, our results suggest that CD1d KO mice, which are NKT cell-deficient mice, are more susceptible to MNV-S7 infection than WT mice. The small intestine of CD1d KO mice has a high MNV-S7 burden for a long period during MNV-S7 infection as results of the impaired production of IFN-γ early in the infection and MNV-S7-specific secretory IgA during the infection. Further research will be needed to examine the immune mechanisms in between NKT cell functions and MNV-S7 infection.

## Declaration of competing interest

The authors declare no conflict of interest in association with the present study.
